# Tremor in Parkinson’s Disease: From Pathophysiology to Advanced Therapies

**DOI:** 10.5334/tohm.712

**Published:** 2022-09-13

**Authors:** Ali H. Abusrair, Walaa Elsekaily, Saeed Bohlega

**Affiliations:** 1 Department of Clinical Neurosciences, Faculty of Medicine, University of Calgary, Calgary, AB, Canada; 2 Division of Neurology, Department of Internal Medicine, Qatif Health Network, Qatif, Saudi Arabia; 3 College of Medicine, AlFaisal University, Riyadh, Saudi Arabia; 4 Movement Disorders Program, Neurosciences Centre, King Faisal Specialist Hospital & Research Centre, Riyadh, Saudi Arabia

**Keywords:** Parkinson’s Disease, Tremor, Levodopa-resistant, DBS

## Abstract

**Background:**

Tremor is one of the most prevalent symptoms in Parkinson’s Disease (PD). The progression and management of tremor in PD can be challenging, as response to dopaminergic agents might be relatively poor, particularly in patients with tremor-dominant PD compared to the akinetic/rigid subtype. In this review, we aim to highlight recent advances in the underlying pathogenesis and treatment modalities for tremor in PD.

**Methods:**

A structured literature search through Embase was conducted using the terms “Parkinson’s Disease” AND “tremor” OR “etiology” OR “management” OR “drug resistance” OR “therapy” OR “rehabilitation” OR “surgery.” After initial screening, eligible articles were selected with a focus on published literature in the last 10 years.

**Discussion:**

The underlying pathophysiology of tremor in PD remains complex and incompletely understood. Neurodegeneration of dopaminergic neurons in the retrorubral area, in addition to high-power neural oscillations in the cerebello-thalamo-cortical circuit and the basal ganglia, play a major role. Levodopa is the first-line therapeutic option for all motor symptoms, including tremor. The addition of dopamine agonists or anticholinergics can lead to further tremor reduction. Botulinum toxin injection is an effective alternative for patients with pharmacological-resistant tremor who are not seeking advanced therapies. Deep brain stimulation is the most well-established advanced therapy owing to its long-term efficacy, reversibility, and effectiveness in other motor symptoms and fluctuations. Magnetic resonance-guided focused ultrasound is a promising modality, which has the advantage of being incisionless. Cortical and peripheral electrical stimulation are non-invasive innovatory techniques that have demonstrated good efficacy in suppressing intractable tremor.

## 1 Introduction

Parkinson’s disease (PD) is a progressive neurodegenerative disorder defined by a constellation of cardinal features that include tremor, bradykinesia, rigidity, and postural instability [1,2]. The spectrum of motor and non-motor manifestations of the disease is further expanding [3]. Tremor is one of the most common motor symptoms in PD and is reported to affect up to 75% of patients during their disease course. Moreover, tremor can be the predominant and most troublesome motor symptom [4,5,6,7]. While several forms of tremor can develop in patients with PD, the typical pill-rolling tremor at rest is the most common [6]. Both kinetic and re-emergent postural forms can also coexist, which may result in substantial functional impairment [6,7,8].

PD is recognised to be heterogeneous, and growing evidence of clinical subgroups has emerged based on the predominant symptom associated with each subtype [9,10,11,12]. In comparison to other PD subtypes, tremor-dominant PD tends to have a slower disease progression, less debilitating non-motor symptoms, decreased probability of developing levodopa-induced dyskinesia (LID), and potential resistance to dopaminergic agents [6,12]. In addition, the response to dopaminergic agents, if any, tends to be higher in resting and re-emergent tremor, whereas the response for kinetic tremor is relatively poor [7,13,14,15]. This phenotype constitutes up to 8% of PD cases as examined in postmortem clinicopathologic studies [16,17].

The purpose of this review is to highlight the underlying pathophysiological mechanisms of tremor in PD and recent advances in therapeutic options.

## 2 Methods

**Figure 1:**
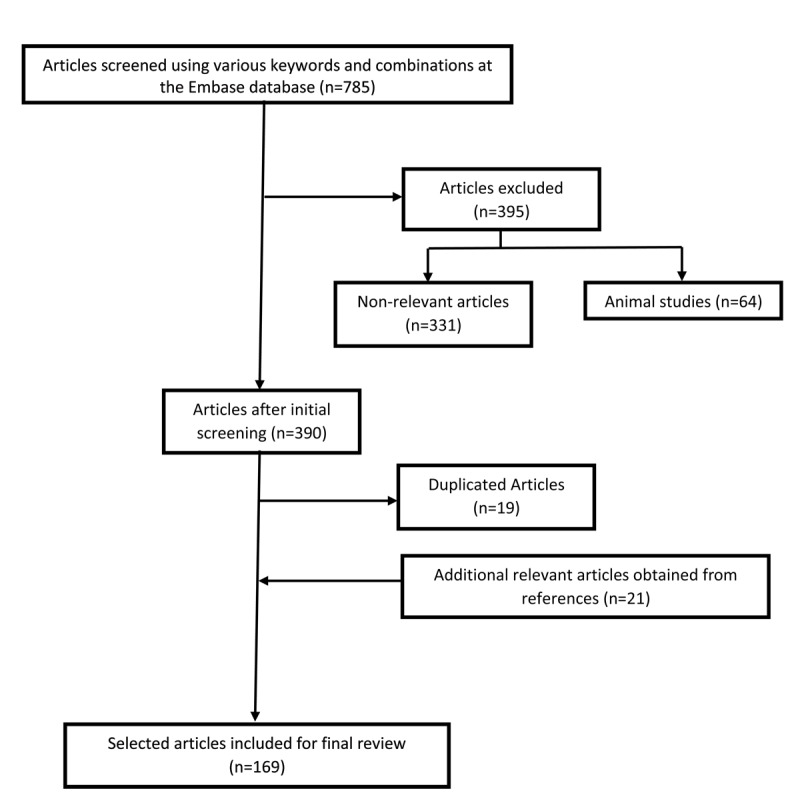
Flow diagram summarizing the steps involved in the literature search.

A structured search of Embase database was conducted, using the following keywords: “Parkinson’s Disease” AND “tremor” OR “etiology” OR “management” OR “drug resistance” OR “therapy” OR “rehabilitation” OR “surgery.” Articles were included if the format was a guideline, original article, review, letter to the editor, or case series. Results from the last 10 years (2012–2022) were prioritized to highlight the most recent advances in pathophysiology and management of tremor in PD. The search included English-language articles only. Articles were excluded if: the subjects were animals, the format was a case report, or the topic was not relevant. Articles were also excluded if there was an overlap between essential tremor (ET) and PD. This resulted in a total of 785 articles. In the final screening process, a total of 169 relevant articles were selected for review (Figure 1).

## 3 Discussion

### 3.1 Pathophysiology

The pathophysiology of tremor in PD is complex and remains incompletely understood. The onset, severity, and progression of tremor are hypothesized to be multifactorial. It is thought to have a distinct pathophysiologic mechanism from classic nigrostriatal dopamine depletion [18,19,20,21].

#### The dimmer-switch model

**Figure 2:**
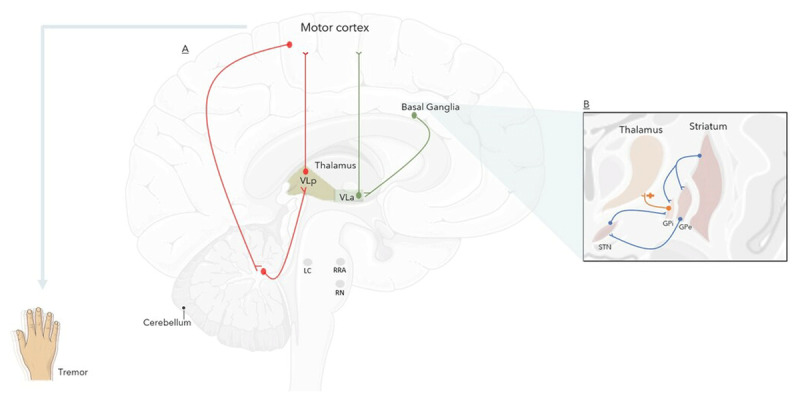
**Cerebral neuronal and neurochemical basis of tremor in Parkinson’s Disease.** The figure shows the main circuits of the dimmer-switch model (A), which includes the cerebello-thalamo-cortical circuit (in red) and the basal ganglia-cortical circuit (in green). The basal ganglia (B) is the key structure that triggers the initiation of tremor. The striatum increases inhibitory output to the globus pallidus internus (Gpi), which in turn stimulates the anterior ventrolateral (VLa) nucleus of the thalamus. This trigger further propagates to the cerebral cortex, where convergence of both circuits occurs. This convergence stimulates the cerebello-thalamo-cortical circuit, which alters tremor amplitude. The figure also shows the main nuclei proposed to have major neurochemical role in tremor pathogenesis: 1. Degeneration in the retrorubral area (RRA) leads to reduced dopaminergic projections to the subthalamic region, the basal ganglia, and the ventrolateral thalamus 2. Reduced serotonergic projections result from degenerative raphe nuclei (RN). 3. Increased noradrenergic projection from the locus coeruleus (LC).

The dimmer-switch model proposes a synchronous oscillatory activity in two separate, but partially overlapping, central pathways [22,23,24]. Cerebello-thalamo-cortical and basal ganglion-cortical loops cause an alteration in normal central neural oscillations and eventually trigger tremor episodes (Figure 2) [22]. This proposed model is based on neurophysiologic, neuroimaging, and intraoperative monitoring studies during functional stereotactic neurosurgical procedures [19,25,26,27]. In addition, stereotactic interventions in anatomic structures related to both pathways (the subthalamic nucleus (STN), ventral intermediate nucleus (Vim), and the internal globus pallidus (GPi)) can suppress tremor, further supporting the role of these structures in the underlying pathogenesis [28]. This model suggests that the basal ganglia is the key structure where a transient activation generates tremor, thus acting the “switch” role [23,24]. First, an oscillatory activity in the striatum causes an increased inhibitory output to the thalamus, which in addition to GPi bursting activity, would generate rhythmic bursting in the thalamic anterior ventrolateral nucleus (VLa) [29]. This eventually projects into the motor cortex where both circuits converge [21,23,26]. The primary motor and premotor cortices are the main areas where this convergence, as well as tremor-related activity, occurs [30,31]. Convergence at this level drives the cerebello-thalamo-cortical circuit, which modulates tremor amplitude, thus acting as the “dimmer” on the switch [31,32]. The role of both circuits was examined through combing functional MRI studies with electromyography (EMG), in which cerebral responses and co-fluctuation can be identified according to any spontaneous variations in tremor amplitude, as peripherally measured with EMG [25,29]. Cerebral activity was found to be time-locked to the onset of high-amplitude tremor episodes and was localized to both the basal ganglia and the cerebello-thalamo-cortical circuit [23]. Furthermore, maximal activity of the basal ganglia structures was detected at the onset of tremor episodes, thus supporting the specific role of the basal ganglia as a driving force for tremor generation, while subsequent tremor amplitude-related activity was localized only to structures related to the cerebello-thalamo-cortical circuit (VLp, cerebellum, and the motor cortex) [23,24]. Patients with tremor-dominant PD were also found to have an increased functional connectivity between the two circuits when compared to non-tremor PD patients, which additionally supports the role of this integrated network model in PD tremor pathogenesis [24].

While the pathophysiologic basis for tremor subtypes in PD remains elusive, insights into the origin of resting and postural components was investigated through non-invasive transcranial magnetic stimulation (TMS) of the primary motor cortex (M1) and the cerebellum. Resetting resting and postural PD tremor can be achieved with M1 stimulation, whereas cerebellar stimulation can reset the postural component only [33,34]. These findings suggest that this cortical area controls the amplitude and the rhythm of resting and postural tremor in PD, while postural tremor modulation is more related to the cerebellum.

#### Role of Dopamine and other neurotransmitters

Degeneration of dopaminergic neurons in the retrorubral area (RRA) of the midbrain, more so than in the substantia nigra pars compacta, may correlate with the generation of tremor in PD [35]. Loss of dopaminergic projections from the RRA to the subthalamic region, the basal ganglia, and the ventrolateral thalamus result in dopamine depletion in these regions and represents one of the main neurochemical bases of tremor generation in PD [30,36,37,38]. Tremor severity was found to correlate with dopamine transporter (DAT) density in the pallidum, while other motor symptoms correlate with DAT density in the striatum [30]. This suggests a more selective pallidal dopamine depletion that leads to basal ganglia dysfunction, which subsequently drives tremor episodes in PD.

In addition to dopamine, other neurotransmitters have been proposed to play a critical role in the pathogenesis of tremor in PD. In patients with tremor-dominant PD, locus coeruleus interneurons have relatively less degeneration, and noradrenaline (NA) receptor binding is increased compared to other PD phenotypes and healthy controls [37,39]. Noradrenaline contribution in parkinsonian tremor is reflected by the effect of cognitive stress, which activates the noradrenergic system, release NA, and result in tremor amplitude aggravation tremor amplitude aggravation [40]. This potential role has also been examined through the administration of intravenous adrenaline in PD patients, which has resulted in an increase in tremor amplitude [24].

The magnitude and severity of tremor are more attributed to serotonin deficiency. Loss of serotonin transporters in the raphe nuclei in the midbrain has been shown to be correlated with more severe tremor [7,19]. Furthermore, 123I-FP-CIT measurement of the median raphe serotonin transporter availability, compared to the putamen dopamine transporter uptake, has shown that more severe tremor scores are correlated with lower raphe/putamen uptake ratio values [41]. In addition, this group of patients tend to have relatively small clinical benefit when receiving acute dopaminergic therapy. Both findings are indicative that more severe, and dopaminergic resistant, tremor are suggestive of more severe raphe nucleus dysfunction [7,41,42].

Due to the impact produced by anticholinergic drugs in PD tremor reduction, acetylcholine has been proposed to contribute to the development of tremor in PD [21,24]. Dopamine deficiency is thought to result in hyperactive striatal cholinergic interneurons, which in turn reduces the release of dopamine and exacerbates PD symptoms, including tremor [19,43].

### 3.2 Phenomenology

The classical tremor in PD is usually asymmetric, predominantly “pill-rolling,” resting tremor of 4–6 Hz frequency that is often suppressed with voluntary movements [8,9,10,44]. It is not uncommon, however, to have the resting component combined with either kinetic and/or postural tremor [44,45,46]. Kinetic tremor is apparent during hand movements such as writing or during finger-to-nose examination. Postural tremor presents while stretching out arms against gravity [45]. Re-emergent tremor is a form of postural tremor, in which a “re-emergence” of tremor appears after a short latency (seconds) when hands are kept in an anti-gravity posture [46]. In the literature, action tremor is usually referred to as either kinetic or postural tremor. Tremor in PD was recently subclassified into four categories based on its phenomenology: Type I, in which tremor is of a pure resting component of 4–6 Hz; Type II, where resting tremor is associated with an action component of similar frequency; Type III, in which patients have an isolated action tremor; and Type IV, where a mixed resting and action tremor coexist, each with variable frequency, and the patient may have features of ET in addition to PD [8].

### 3.3 Management

#### 3.3.1 Pharmacotherapy

**Figure 3:**
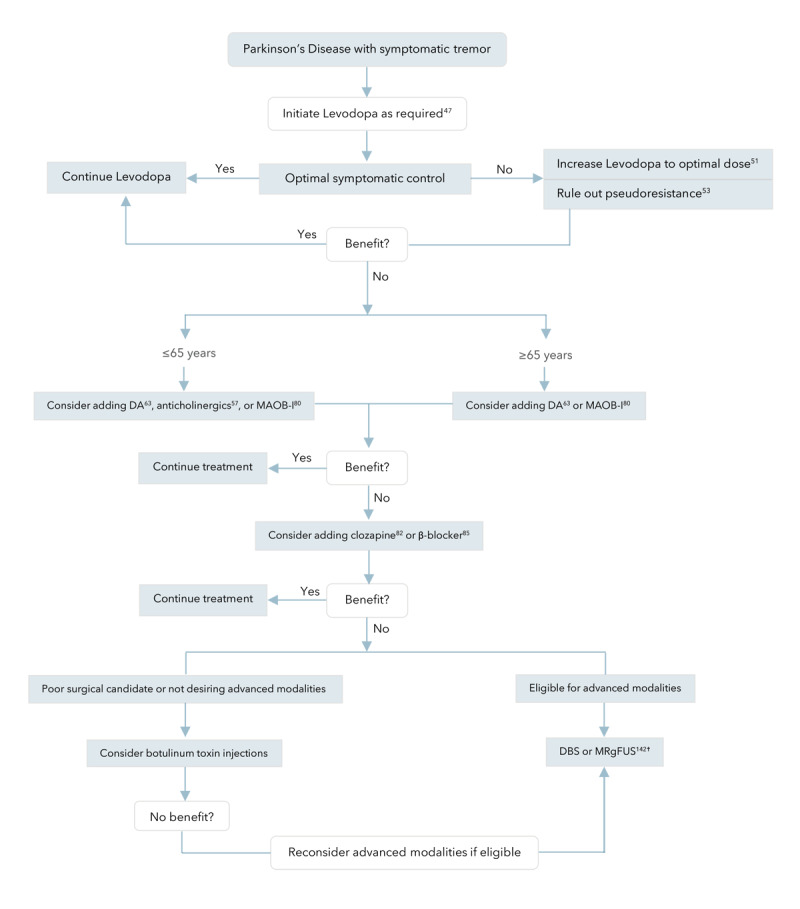
**Algorithm for the treatment of Parkinson Disease with predominant symptomatic tremor.** ^†^ No strong evidence to support long term, sustained efficacy, and safety. Currently, the modality is mostly applied within the scope of clinical trials and registries.

Levodopa and other dopaminergic agents remain the first-line therapeutic option for all motor symptoms in PD, including tremor [47,48,49,50,51,52]. However, the choice of the optimal agent might be driven or limited by individualized factors. Disease-related characteristics, like tremor severity and sensitivity to levodopa, in addition to patient-related factors such as age, functional and cognitive status, can guide the choice of pharmacotherapy (Table 1; Figure 3) [47,51].

**Table 1:** Pharmacotherapeutic options in the treatment of Parkinson’s disease tremor.


MEDICATION	MECHANISM OF ACTION	STARTING DOSAGE (MG)	TITRATION	MAXIMUM (MG)	SIDE EFFECTS	COMMENTS

Levodopa	Metabolic precursor of dopamine	Levodopa-carbidopa 100/25 mg TID	Increase by 1–2 tablets every week	1200–1500 mg/day in 3–4 divided doses	Nausea, vomiting, postural hypotension, confusion or hallucinations	Dose and frequency can be increased as tolerated

		Levodopa-benserazide 100/25 mg TID				

Dopamine Agonists	Stimulate dopamine receptors	Pramipexole 0.125 mg TID	Slow titration every 5–7 days	4.5 mg/day	Somnolence, constipation, dizziness, hallucinations, sleep attacks and ICD.	

		Pramipexole ER 0.375 mg TID				

		Rotigotine transdermal patch 2 mg/24 hours	May increase by 2 mg/24 hours at weekly intervals	8 mg/24 hours		

		Ropinirole 0.25 mg TID	Slow titration at weekly intervals	24 mg/day		

		Ropinirole ER 2 mg OD				

MAOB-I	Inhibits monoamine oxidase enzyme	Selegiline 2.5–5 mg OD	Slow titration at weekly intervals	10 mg/day in 2 divided doses	Headache, dizziness, insomnia, nausea	
	
		Rasagiline 0.5 mg OD		1 mg/day		

Anticholinergics	Antagonise the effects of acetylcholine at muscarinic receptors postsynaptic to striatal interneurons	Benztropine 0.5mg/day	Increase by 0.5 mg every 5–7 days	6 mg/day in 2 to 4 divided doses	Memory impairment, confusion, and hallucinations plus peripheral antimuscarinic side effects.	Rapid withdrawal can result in exacerbation of parkinsonism

		Trihexyphenidyl 1 mg/day	Gradual increase by 2 mg at 3–5 days interval	12–15 mg/day in 3 to 4 divided doses		

Clozapine	Has anticholinergic and anti-serotonergic properties	12.5 mg	Add 12.5mg every 1 to 2 weeks	75–100 mg/day	Agranulocytosis, sedation, hypotension, hypersalivation and fever have been reported.	Requires routine blood monitoring for blood count

Clonazepam	Enhances GABA activity	0.5mg OD	Increase by 0.5 mg every 3–4 days	6 mg/day	sedation, memory loss and confusion.	

Propranolol	β1- and β2-receptor blocker	**Regular** 10–20 mg BID	Titration at 3–7 days interval	320 mg/day	Sedation, insomnia, depression, hypotension, diarrhea, constipation and impotence	Rapid withdrawal can result in arrythmias

		**ER** 60–80 mg OD				


GABA: gamma-aminobutyric acid; OD: once daily, BID: twice per day. TID: three times per day; ER: Extended Release; ICD: Impulse Control Disorder.

##### Levodopa

The effect of levodopa is known to be greatest for bradykinesia, while the effect on tremor control is relatively variable [50,52,53,54]. Favorable effects are more pronounced for resting and re-emergent components compared to kinetic or pure postural tremor [46,55]. Overall, it ranges widely from no objective clinical response to 80% tremor reduction [56,57]. This inconsistent response has led to the proposed classification of tremor into three subtypes: dopamine-responsive, dopamine-resistant, and a partially overlapping intermediate group [58,59,60]. The dose and duration required to consider tremor as dopamine-resistant have not yet been determined. Based on this classification, dopamine-resistant was defined according to its response during a levodopa challenge test (LCT), in which higher doses of levodopa are tested in an OFF state. Patients with dopamine-resistant PD tremor demonstrate a lack of clinical and electrophysiologic response despite receiving up to double their usual dose of levodopa during the test [59].

Poor response in some patients might be attributed, in part, to pseudoresistance, a phenomenon in which levodopa sensitive symptoms falsely appear to be resistant [15,52,60]. This occurs in the context of multiple underlying mechanisms that contribute to a suboptimal response [60]. Factors leading to pseudoresistance include gastrointestinal dysfunction causing poor absorption or high protein diets interfering with levodopa. Moreover, cognitive stress is a known factor that attenuates the therapeutic effect of dopamine [24,52,60].

As the effect of levodopa is expected to be dose-dependent for tremor control, a dose increase might eventually be required, even if other symptoms show a robust response to lower doses [61]. However, the main challenge remains related to the maximum dose, which may be limited because of the potential dose-related side effects like nausea, vomiting, dyskinesias, or hallucinations. Furthermore, some studies have suggested that tremor might be worsen with a higher levodopa dosage [62].

##### Dopamine Agonists (DAs)

Pramipexole, apomorphine, and other DAs can augment the effect of levodopa, hence, providing greater tremor control [63,64]. They can be used as an initial monotherapy, or as adjuvant addition to levodopa [50,65]. Both pramipexole and pergolide produce a similar degree of resting tremor suppression when used as monotherapy [66]. The addition of pramipexole to levodopa results in an estimated 45% reduction in the Unified Parkinson’s Disease Rating Scale (UPDRS) tremor scores and significantly lower tremor occurrence during waking hours as recorded by long-term electromyography (EMG) [63]. Apomorphine is a potent, relatively short-acting DA that can be administered with a continuous subcutaneous infusion pump, or an intermittent sublingual, and subcutaneous injection [67,68]. Apomorphine can provide a comparable effect on tremor produced by levodopa, but with a considerably lower mean duration of effect [69,70].

Levodopa and DAs have comparable dopaminergic side effect profiles [49,69]. An additional dose-dependent side effect linked to DAs is the development of impulse control disorder (ICD), which is estimated to have up to a 50% five-year cumulative incidence risk [71]. Treatment of early PD with DAs can be associated with a reduced risk of motor fluctuations in the first five years after initiation, especially with younger patients [72,73].

##### Anticholinergics

Anticholinergic medications, including trihexyphenidyl and benztropine, can be considered if tremor control is inadequate with dopaminergic agents [15,74]. Anticholinergics are effective in improving PD tremor and other motor symptoms, but with a high risk of neuropsychiatric and cognitive adverse events [75]. These factors have limited the use of anticholinergics as they were a common reason for non-compliance. In addition, outcome measures of these agents vary widely. A significant improvement in tremor was found in some studies, while others have shown poor tremor response, but with improvement in bradykinesia and rigidity [75]. Due to the potential adverse effects, anticholinergics should only be used for tremor-dominant PD patients who are young and have failed to improve with dopaminergic agents [15,74]. It is important to utilize a slow taper, if required, as rapid discontinuation may manifest with acute exacerbation of parkinsonism [76].

##### Monoamine Oxidase B (MAO-B) Inhibitors

MAO-B inhibitors act by increasing the bioavailability of central monoamines, including dopamine. They can be very effective at improving motor and non-motor symptoms in the early stages of PD, which might delay the need for levodopa [77]. The ameliorating beneficial effects of MAO-B inhibitors on motor symptoms are notably greater in akinetic/rigid PD compared to tremor-dominant PD [78]. However, rasagiline was selectively studied as a monotherapy, or as an adjuvant therapy to levodopa in patients with tremor-dominant PD, and found to have a significant effect on tremor reduction as early as 10 weeks from treatment initiation [79,80].

##### Clozapine

Clozapine is an antipsychotic agent that is commonly used in the treatment of schizophrenia and drug-induced psychosis [81]. The exact mechanism by which it exerts its anti-tremor effect is not fully understood but may be attributed to its anticholinergic and antiserotonergic properties [82]. Resting and postural tremor can be reduced in up to 72% of PD patients, and tremor scores can be reduced by 64% [83]. In addition to its anti-tremor effect, the advantage of its anti-psychotic action might be of significance in patients experiencing psychosis [82]. One major limitation of clozapine is the risk of developing agranulocytosis, which mandates frequent blood monitoring [84].

##### Beta-blockers

Propranolol is a non-selective beta-blocker that has been widely used in the treatment of ET. In PD tremor, the use of propranolol and other beta-blockers lacks evidence to determine efficacy and safety [85]. Propranolol can improve the postural component of PD tremor, and it is clinically useful in the context of associated anxiety and stress that aggravate tremor [86]. However, the efficacy is usually not sustained, and a large proportion of patients eventually discontinue the medication because of rapid tolerance, loss of initial response and the increased risk of orthostatic hypotension [85]. Like anticholinergics, beta-blockers should be tapered gradually to prevent withdrawal symptoms [87].

##### Other agents

Zuranolone is a novel gamma-aminobutyric acid (GABA) receptor positive allosteric modulator that improves tremor scores by 40% when used as an adjuvant agent with dopaminergic therapies [88]. Other therapeutic options include clonazepam, budipine, zonisamide, amantadine, and mirtazapine, all of which have shown variable degrees of nonsustained tremor control [65].

#### 3.3.2 Botulinum toxin injections

Botulinum neurotoxins (BoNT) are proteins derived from the bacterium *Clostridium botulinum*. They act at the cholinergic presynaptic nerve terminals by cleaving and inactivating SNARE proteins and subsequently inhibiting the release of acetylcholine. This, in turn, prevents muscle contraction and results in paralysis of injected skeletal muscles. In addition, BoNT blocks gamma motoneurons and reduces muscle spindle afferent input to the central nervous system [89,90,91,92]. BoNT type A (BoNT-A) has been widely used to treat tremor and other movement disorders, and it can be a rescue option for patients who for patient who have pharmacologically-refractory tremor and considered poor candidates for advanced therapies [90,91]. The reported success rate is variable and is influenced by factors such as dose, muscles selected, technique, and provider experience [92,93,94]. The Yale Technique and Sensor-Based Kinematics have been proposed as safe and supportive methods that can enhance efficacy [95]. The Sensor-Based Kinematics method uses motion sensors to analyze angular tremor amplitude, which provides better individualized muscle selection [94,95]. The Yale Technique uses EMG guidance of determined muscles to further enhance accurate muscle selection and success rate [95].

Forearm flexors and extensors are traditionally targeted muscles. A fixed, initially low, BoNT dose is suggested to avoid dose-dependent weakness [91,92,95]. Forearm flexors are prioritized over extensors because of the relatively higher rate of extensor finger weakness [96,97]. The long-term effect of BoNT injections was demonstrated with a mean follow-up duration of 29 months, with over 80% of patients reporting moderate or marked improvement at their first and last visits [92,98]. The mean UPDRS scores for resting and kinetic tremor were significantly reduced when compared to baseline when BoNT injections were coupled with kinematic guidance [98].

#### 3.3.3 Advanced therapies (Table 2)

**Table 2:** Advanced surgical modalities for Parkinson’s Disease tremor.


MODALITY	SELECTION CRITERIA	TARGETS	ADVERSE EVENTS

A. DBS	- Diagnosis of IPD with ≥ five-years disease duration - Age ≤ 75* - Medication-refractory symptoms or fluctuations - Dopaminergic responsiveness confirmed by LCT** - Intact cognitive status - No intracranial pathology on neuroimaging	STN, GPi, Vim, PSA	Cognitive decline, cerebral hemorrhage, infection, hardware failure, delayed lead migration, and death

B. Lesioning therapies			

MRgFUS	- Diagnosis of IPD - Medication-refractory symptoms or fluctuations - Intact cognitive status - No intracranial pathology on neuroimaging - No history of DBS or prior stereotactic ablation - No bleeding liability - Skull density ratio ≥0.45	Thalamotomy, Subthalamotomy, Pallidotomy	Headache, dizziness and vertigo, transient ataxia, paresthesia, and weakness
	
GK		Thalamotomy, Subthalamotomy	Transient paresthesia and hemiparesis, dysphagia, and death
	
RF		Thalamotomy	Transient paresthesia, hemiparesis, dysarthria, ataxia, confusion, cognitive decline, and intracerebral hemorrhage


IPD: Idiopathic Parkinson’s Disease; * No consensus agreement; ** May not be reliable indicator in the case of tremor-dominant PD.

##### Deep Brain Stimulation (DBS)

In the last three decades, deep brain stimulation (DBS) has risen as the most common advanced surgical modality in PD [99]. This is due to its long-term efficacy in improving refractory and poorly-controlled motor symptoms and fluctuations [100,101,102,103,104]. Candidacy for DBS is based on a detailed evaluation, which incorporates cognitive status, responsiveness to levodopa in the LCT, and the nature of associated symptoms and motor fluctuations [105,106]. A cutoff of 30% improvement in the UPDRS III during the LCT was established as a threshold for levodopa and potential DBS responsiveness. However, patients with dopamine-resistant tremor might show a poor response in the LCT [59,107,108,109]. Hence, the LCT may not be a reliable indicator for candidacy and repressiveness to DBS in the case of refractory PD tremor [110,111]. DBS electrodes replace and mimic the therapeutic effect produced by lesioning therapies but without inducing significant brain lesions, which provides the advantage of reversibility. Furthermore, DBS can be applied to both cerebral hemispheres, an option currently limited to one side only in lesioning therapies [119,120].

The therapeutic mechanism of DBS is not well understood [112,113,114]. Electrode placements in structures with neural oscillatory activity (i.e., the basal ganglia and cerebello-thalamo-cortical loops) could disrupt this oscillatory activity and, hence, alter tremor generation and/or amplitude [115,116]. Several imaging studies have investigated patterns of metabolic changes before and after DBS electrode placement. Patients with tremor-dominant PD display a distinct pattern, a tremor-related metabolic pattern (PDTP), which correlates with tremor severity and is characterized by an increased activity in the cerebellar dentate nucleus and primary motor cortex. In non-tremor dominant PD, a pattern with hypermetabolism in the pons, globus pallidus, and the thalamus can be seen (PD-related metabolic pattern: PDRP), and it correlates with the severity of other motor symptoms [115]. Interestingly, Vim DBS can only reduce PDTP activity, while STN and GPi DBS can reduce both PDTP and PDRP activities [117,118]. These findings explain why such targets would improve all motor symptoms, including tremor, while Vim selectively improves tremor only.

DBS target selection for patients with tremor-dominant PD is individualized. Placement of electrodes in the STN, Vim, GPi, and the posterior subthalamic area (PSA) are reportedly effective in alleviating PD tremor [121,122,123,124]. However, STN and GPi have an apparent benefit compared to other targets as they improve all motor symptoms [121]. Both targets have comparable efficacy and can reduce resting and kinetic tremor components [124]. Interestingly, STN-DBS can achieve better outcomes for arm tremor compared to chin and lower extremity tremor [125]. STN-DBS may be superior to GPi-DBS for controlling dopamine-resistant tremor [126]. Compared to other targets, Vim-DBS is associated with better improvement in UPDRS tremor scores in the off state, which would allow for greater medication reduction [121]. This factor would arguably favor Vim over other targets for PD tremor. However, the evolution of other PD symptoms and motor fluctuations can pose a challenge, as Vim is not the preferred target [127,128]. Therefore, selecting Vim should be reserved for patients with a long-standing, mostly unilaterally-dominant tremor as the main symptom in the absence of other motor features or fluctuations. Dual implantation of the GPi and Vim can be applied for patients with dopamine-resistant tremor, who have other motor symptoms or fluctuations that would benefit from GPi stimulation [129].

The long-term efficacy of DBS is well-documented for treating motor complications of PD and maintaining improvement in quality of life (QoL) [130,131,132]. Both STN and GPi targets can provide a relatively comparable, persistent benefit in the first few years after electrode implantation [133,134,135]. For GPi-DBS, fewer patients exhibited cognitive decline, gait disorders, or speech difficulties compared to STN-targeting DBS [134]. However, additional, long-term benefits in core motor symptoms are more consistent with STN-targeting DBS [132]. Bilateral Vim-DBS is potentially associated with dysarthria, loss of balance, and incoordination over the long term [136,137].

##### Lesioning Therapies (LTs)

LTs can successfully be utilized for the treatment of PD motor symptoms and are considered one of the most effective therapies for the management of refractory tremor in PD [138]. MRI-guided focused ultrasound (MRgFUS), Gamma Knife (GK), and radiofrequency (RF) thermoablation are the main and available LT modalities in practice [138,139,140,141].

Although LTs originally fell out of favor with the advancement of DBS, there is a growing interest in LTs in recent years with the introduction of incisionless therapies like MRgFUS or GK [139]. Both therapies have the advantage of not requiring general anesthesia, and compared to DBS, have fewer side effects related to surgical interventions [140]. In addition, LTs might offer alternative surgical options to DBS for underserved and remote areas where resources and distance can limit ongoing treatment and monitoring. Both MRgFUS and RF thermoablation have the advantage of providing a real-time assessment of the benefit during the procedure before reaching a final, clinically-based lesioning [139]. Compared to the immediate results of other modalities, the benefits and adverse effects related to GK lesioning are expected to develop several months after the procedure [141]. Unlike DBS, lesioning therapies produce permanent lesioning, which is considered a major drawback. Furthermore, evidence of the long-term efficacy of DBS in PD is immense compared to the relatively small and short-term investigational data addressing the efficacy and safety of LTs [100,130,131,142].

##### MRI-guided focused ultrasound (MRgFUS)

MRgFUS has recently emerged as a very promising therapeutic option for refractory tremor in PD. Safety and efficacy were first demonstrated in the treatment of ET, leading to its Food and Drug Administration (FDA) approval as a rescue management modality for medication-refractory tremor [126]. While available data is encouraging and has confirmed an overall improvement in tremor scores and QoL in PD, there is a lack of sufficient/high quality evidence to suggest the regular use of MRgFUS in PD with refractory tremor [142].

MRgFUS lesioning is conducted through minimally invasive thermal ablation with phased-array transducers, which enable precise, incisionless transcranial delivery of acoustic energy [123]. Like ET, the most common examined anatomic target in PD tremor is the Vim nucleus of the thalamus [139,143,144]. In one RCT, STN was the main target, while other small case series have studied the pallido-thalamic tract (PTT) and GPi [145]. MRgFUS thalamotomy can achieve an estimated improvement in the clinical rating scale for tremor sub-scores by a median of seven points, as well as in on-medication median UPDRS motor scores by eight points when compared to pre-intervention [146,147]. As with thalamotomy, MRgFUS subthalamotomy was also found to achieve improvement in the UPDRS-III (including tremor scores), QoL, and ADLs as measured in the UPDRS-II [146]. MRgFUS of the PTT has been found to be as safe and effective, and up to 88% of mean tremor reduction has been achieved with this target [148].

Currently, the modality is applied mostly for unilateral lesioning to control the most affected side [139]. Staged bilateral lesioning remains controversial, with a growing number of reports, only in ET, showing good overall efficacy and a similar safety profile to unilateral lesioning [149].

Most side effects are transient, usually subsiding by three to 12 months after the procedure [150]. The most common procedural side effects are headache, dizziness, and vertigo. Ablation-related side effects include transient ataxia, paresthesias, and weakness [143,144,145,146,147,148,149,150]. Additionally, MRgFUS subthalamotomy can result in dyskinesia in the off-medication state in up to 22% of patients, which can persistent up to three months [145]. Compared to DBS, cognitive decline appears to be minimal and tends to be limited to verbal fluency and inhibition [144].

##### GK Thalamotomy

GK radiosurgery is an incisionless lesioning procedure in which high-dose radiation is applied to pre-specified brain targets [139,151,152]. The modality is dependent on pre-procedural, imaging-based planning [154]. The absence of real-time targeting may result in an unpredictable effect. Most reports on GK radiosurgery have a small sample size, and no randomized trials have addressed its efficacy compared to other modalities [153]. GK lesioning is reported to achieve improvements of UPDRS tremor items by 71% and 60% at 12 and 52 months, respectively [154]. Patients report 88% complete or near-complete alleviation of PD tremor [151,153]. GK thalamotomy could be a preferred option for patients with advanced age or associated comorbidities who are not candidates for DBS. Adverse events are generally rare and usually transient [140]. However, serious events like thalamic hemorrhage have been reported [155].

##### RF Thermoablation

Unlike MRgFUS and GK treatments, RF ablation is performed through a frontal burr hole of the skull and requires brain penetration with a special electrode [156,157]. The electrode can be heated to sub-ablative thermal temperatures to produce a “test lesion.” Subsequently, a higher temperature is applied to produce a permanent lesion at the desired target [139]. RF ablation was the modality of choice for tremor in PD before the introduction of DBS in the late 1980s. Cost is relatively lower than other modalities [139]. Lesioning through RF thermoablation in PD is usually implemented through targeting the GPi, the thalamus, or the STN. The modality seems to achieve the highest tremor control in PD when targeting the thalamus, with improvements reaching up to 74%. In most cases, the use is limited to one side, as bilateral RF thalamotomy is typically associated with a high rate of adverse effects [156,157]. Complications are usually transient and result from local edema produced after ablation, which ultimately recover as the edema resolves [139]. One of the main concerns regarding the procedure is the potential risk of intracerebral hemorrhage and subsequent neurological deficits. These could occur at the entry point, in the electrode path, or at the final ablation site [157].

##### Levodopa/Carbidopa Intestinal Gel (LCIG)

LCIG is administered continuously by a portable pump via a percutaneous endoscopic gastrojejunostomy (PEG-J) tube [158]. LCIG provides a more stable plasma concentration in patients with poorly controlled motor symptoms or fluctuations in advanced PD [159]. The modality is considered an optimal option for those excluded from surgical interventions who require sustained dopaminergic therapy for refractory motor symptoms. The improvement is reflected in the UPDRS part III, including tremor sub-scores. A complete resolution in resting tremor after 12 months of LCIG treatment was reported in up to 78% of patients who had baseline resting tremor pre-treatment [160]. No current evidence to support the role of LCIG in patients with tremor-dominant PD.

#### 3.3.4 Physical therapy and rehabilitation

Physical therapy and intense rehabilitation can improve various motor and non-motor aspects of PD, in addition to their potential long-term effect in slowing disease progression [166,167,168]. Specialized rehabilitation techniques, like aerobic and resistance exercises, have demonstrated improvement in global motor functions. Among resistance exercises, eccentric-based exercises have specifically shown a favorable effect in improving tremor [168]. The efficacy is noted on tremor amplitude at rest, with no clear benefit on postural or kinetic tremor. Resting tremor amplitude decreased by 56% in participants who went through eccentric-based exercise sessions [167,168]. In addition, hand movement and cycling exercises are additional and effective methods for reducing tremor amplitude and frequency [168].

Portable assistive devices have been found to enhance Activities of Daily Living (ADL) and QoL [169]. Liftware Steady and Gyenno Spoon can significantly improve handling utensils. Other limb weights and handheld devices might be optimal for handwriting.

#### 3.3.5 Non-invasive cortical and peripheral electrical stimulation

Non-invasive stimulation techniques are emerging new modalities for tremor reduction. It is regarded as complementary methods for treating tremor in PD via tailored central or peripheral stimulation [161]. In practise, neither modality is frequently used or readily available.

The therapeutic effect of cortical stimulation has been observed through the application of high-frequency repetitive transcranial magnetic stimulation (rTMS) and anodal transcranial direct current stimulation (tDCS) [161]. Both techniques act by identifying the timing of cortical oscillations, followed by stimulating the motor cortex to induce phase cancellation of the rest-tremor rhythm [162]. Slow alternating periods of phase cancellation, with stimulation delivered at these specified phase alignments, demonstrate controlled suppression of the ongoing tremor. Improvement of the UPDRS-III baseline motor scores, along with tremor reduction, has been achieved in 50% of patients [163].

Peripheral electrical stimulation can suppress tremor through three modalities: functional electrical stimulation (FES), sensory electrical stimulation (SES), and transcutaneous electrical nerve stimulation (TENS) [164]. The FES method performs the best in tremor attenuation. FES induces muscle contraction to modulate its intrinsic property for suppressing tremor [165]. Surface EMG can be used to assess tremor reduction, and further adjust FES if required, without affecting voluntary movements [166]. Outcomes vary widely from 7% to 90% reductions in tremor amplitude [164].

## 4 Conclusion

Tremor is one of the most common symptoms associated with PD. The complexity of PD tremor and the wide and unpredictable response to therapeutic modalities remain challenging. Poor response to dopaminergic agents is common, reflecting the role of multiple underlying pathophysiologic processes. Evidence for advanced modalities is heterogeneous, with no sufficient comparative studies to address their efficacy in this specific group of PD patients. Despite promising results, long term data of newer advanced modalities, like MRgFUS, shall be sought to ensure safety and sustained efficacy.

## Disclosures

Parts of Figure 2 were drawn by using pictures from Servier Medical Art. Servier Medical Art by Servier is licensed under a Creative Commons Attribution 3.0 Unported License (https://creativecommons.org/licenses/by/3.0/).
